# The Substitution of a Lead Plate for a Portion of the Frontal Bone

**Published:** 1880-09

**Authors:** M. H. Post

**Affiliations:** St. Louis


### THE SUBSTITUTION OF A LEAD PLATE FOR A PORTION OF THE FRONTAL BONE.

BY M. H. POST, M. D., ST. LOUIS.

THE following case is one of interest from its rarity, and from its unexpected, favorable result. When I took the case under consideration, I was unable to find a record of any similar case; and I hope my desire to help some professional brother in a similar strait, is a sufficient excuse for presenting the following account:

Some two years ago, while assistant physician to the St. Louis Female Hospital, a patient came under my immediate charge, suffering with tertiary syphilis. She had passed through the preceding stages, and during part of the time had suffered great pain, and had acquired the opium habit, at times taking as much as a drachm of morphine a day. One night, while under the influence of morphine, she struck her head against a nail (this is the account given me, but I think it doubtful, as there are number of scars on her forehead), which resulted in necrosis of the bone.

When she came under my treatment, she had several gummata, and an ulcer upon her forehead, exposing the bone. She was put on as large doses of potassium iodide as she could bear. She was cured of the morphine habit; and the necrosed bone, separating from the living bone, was removed. After being in the hospital for a number of months, she recovered, and was discharged.

During the month of April, 1879, the woman came to my office to know if I would not do something to improve her personal appearance. At that time her health was good; but there was a deep depression near the center of her forehead, where the loss of bone had occurred. The depression was about $\frac{1}{6}$ inch deep, ¾ inch in its transverse axis, ⅞ inch in its vertical axis; approximately rectangular. It was noticeable at some distance, and was too low down to be covered with her hair. She wished me to fill it up in some way. I tried to dissuade her, telling her that any thing introduced beneath the skin would irritate, and ultimately ulcerate out, making a larger scar than the original one. I consulted with several medical gentlemen about the case, and they all advised me to leave it alone; and I was very sorry when the patient reappeared. I told her the chances were nine out of ten against success; but she insisted, and agreed to take the risk. Accordingly, June 24th, 1879, with the assistance of Dr. McCandless and Dr. F. Glasgow, I performed the operation. The day previous I took a cast of the depression in plaster of Paris, from which I made a lead plate. My reasons for using lead were that the tolerance of bullets in the body seemed to teach that lead is innocuous; lead was much cheaper than silver, and at the last moment could be cut into a new shape, if necessary. The patient had been taking potassium iodide for some days.

As soon as the patient was anesthetized, I made a horizontal incision about half an inch above the upper margin of the depression; through this cut I dissected up the skin and scar tissue, keeping close to the bone. When the depression was reached, there was considerable difficulty, as the scar tissue was extremely thin, and firmly adherent to the bone. The dissection having been accomplished, the lead plate was slipped in, and the horizontal incision sewed up. The knives used and the plate were immersed in a weak solution of carbolic acid in water. The plate weighed a drachm and a half. The wound was dressed with cold water dressings. There was considerable redness and heat following the operation, and considerable serum was effused about the plate, so much that twice I drew it off with a hypodermic syringe. The case progressed favorably, and

I find in my note book, "July 28th, the forehead is smooth, and there are no signs of inflammation."

A bandage was worn about the forehead for some time to keep the plate from moving about, and it also was pleasant to the patient, as it seemed to relieve a sense of weight which the plate produced. This has been given up, and there are no signs of the foreign body doing any harm, it having been tolerated for over a year.

There are three points particularly to be borne in mind—1st, the plate lies on bone; 2d, it is covered by scar tissue; and 3d, the scar tissue is not more than $\frac{1}{16}$ inch thick.

I saw the patient to-day (June 30th, 1880); the plate was in place, giving no trouble, and was filling its purpose so well, that I found myself examining the wrong portion of the forehead.

